# Biphasic effect of extracellular ATP on human and rat airways is due to multiple P2 purinoceptor activation

**DOI:** 10.1186/1465-9921-6-143

**Published:** 2005-12-08

**Authors:** Boutchi Mounkaïla, Roger Marthan, Etienne Roux

**Affiliations:** 1 Laboratoire de Physiologie Cellulaire Respiratoire, Université Bordeaux 2, Bordeaux, F-33076 France; Inserm, E356, Bordeaux, F-33076 France

## Abstract

**Background:**

Extracellular ATP may modulate airway responsiveness. Studies on ATP-induced contraction and [Ca^2+^]_i _signalling in airway smooth muscle are rather controversial and discrepancies exist regarding both ATP effects and signalling pathways. We compared the effect of extracellular ATP on rat trachea and extrapulmonary bronchi (EPB) and both human and rat intrapulmonary bronchi (IPB), and investigated the implicated signalling pathways.

**Methods:**

Isometric contraction was measured on rat trachea, EPB and IPB isolated rings and human IPB isolated rings. [Ca^2+^]_i _was monitored fluorimetrically using indo 1 in freshly isolated and cultured tracheal myocytes. Statistical comparisons were done with ANOVA or Student's *t *tests for quantitative variables and χ^2 ^tests for qualitative variables. Results were considered significant at P < 0.05.

**Results:**

In rat airways, extracellular ATP (10^-6^–10^-3 ^M) induced an epithelium-independent and concentration-dependent contraction, which amplitude increased from trachea to IPB. The response was transient and returned to baseline within minutes. Similar responses were obtained with the non-hydrolysable ATP analogous ATP-γ-S. Successive stimulations at 15 min-intervals decreased the contractile response. In human IPB, the contraction was similar to that of rat IPB but the time needed for the return to baseline was longer. In isolated myocytes, ATP induced a concentration-dependent [Ca^2+^]_i _response. The contractile response was not reduced by thapsigargin and RB2, a P2Y receptor inhibitor, except in rat and human IPB. By contrast, removal of external Ca^2+^, external Na^+ ^and treatment with D600 decreased the ATP-induced response. The contraction induced by α-β-methylene ATP, a P2X agonist, was similar to that induced by ATP, except in IPB where it was lower. Indomethacin and H-89, a PKA inhibitor, delayed the return to baseline in extrapulmonary airways.

**Conclusion:**

Extracellular ATP induces a transient contractile response in human and rat airways, mainly due to P2X receptors and extracellular Ca^2+ ^influx in addition with, in IPB, P2Y receptors stimulation and Ca^2+ ^release from intracellular Ca^2+ ^stores. Extracellular Ca^2+ ^influx occurs through L-type voltage-dependent channels activated by external Na^+ ^entrance through P2X receptors. The transience of the response cannot be attributed to ATP degradation but to purinoceptor desensitization and, in extrapulmonary airways, prostaglandin-dependent PKA activation.

## Background

ATP is an extracellular messenger released by different cells that modulate lung functioning. ATP can be liberated from parasympathetic nerves as co-transmitter with acetylcholine [1], from epithelial cells [2], for example following exposure to air pollutants [3], and is released, probably from cell lysis, during lung injury [4]. ATP stimulates surfactant production by type II pneumocytes [5], Cl^- ^secretion by epithelial cells and the activity of the mucociliary escalator [6]. ATP also acts on airway smooth muscle (ASM) cells, inducing ASM cell proliferation [7] and changes in airway contractility [8].

Receptors for ATP are classified into 2 families. P2X receptors are ionotropic receptors that, upon activation by ATP, initiate extracellular Ca^2+ ^and Na^+ ^influx. P2Y receptors are 7-transmembrane domain receptors that are coupled to G-proteins. When stimulated, they activate PLC leading to inositol 1,4,5-trisphosphate production and intracellular Ca^2+ ^release via G_q/11 _protein, or modulate cAMP production and PKA activity via G_s _or G_i _binding [9,10].

It has been shown that extracellular ATP modulates cytosolic Ca^2+ ^response and contraction in a variety of smooth muscle. However, its effect on airway smooth muscle reactivity has not been comprehensively investigated and the results are quite controversial. In normal rat, intratracheal instillation of ATP *in vivo *increases airway resistance [11]. In lung slides obtained from isolated mouse lung, Bergner and co-workers have shown that ATP induced a transient contraction and cytosolic Ca^2+ ^oscillations mediated by P2Y purinoreceptors, but has no effect on acetylcholine-induced contraction [8]. By contrast, Aksoy and Kelsen [12] have shown in isolated rabbit tracheal strips that ATP alone did not produce any contraction but rather induced relaxation on strips precontracted with acetylcholine, a mechanical response due to P2 receptor activation. A relaxant effect on precontracted isolated rings has also been reported in guinea-pig trachea, but this effect was attributed to P1 receptor stimulation [13].

When present, the contractant effect of ATP alone seems to be associated with [Ca^2+^]_i _increase. Bergner and co-workers reported, in mouse freshly ASM cells, that ATP induced an oscillating [Ca^2+^]_i _response [8], while Michoud and co-workers observed in cultured rat trachea cells a non oscillating [Ca^2+^]_i _response [14]. Both authors attributed the [Ca^2+^]_i _response to intracellular Ca^2+^, whereas in pig cultured ASM cells, Sawai and co-workers showed that the ATP-induced [Ca^2+^]_i _response was decreased in the presence of extracellular Ca^2+ ^[15,16].

The aim of this study was therefore to characterize the effect of extracellular ATP on airway reactivity. Since results obtained in airways with different calibres suggest that it may act differentially along the airway tree, we compared the effect of ATP in rat trachea, extrapulmonary bronchi (EPB) and intrapulmonary bronchi (IPB) and, additionally, in human IPB. We have investigated whether ATP modulation of airway reactivity was due to an indirect or direct action on airway smooth muscle cells. We have also determined the pharmacological profile of the receptors involved in the ATP-induced response and the subsequent intracellular pathways, and, finally, we have assessed the implication of enzymatic ATP degradation in the response pattern to purinergic stimulation.

## Methods

### Preparation of rat tissues

Rat airways were obtained from male Wistar rats 10–15 weeks old, weighing 300–400 g. Animals were treated and sacrificed according to national guidelines, with approval of the local ethical committee. For each experiment, a rat was stunned and killed by cervical dissociation. Heart and lungs were removed in bloc, and the trachea, the extracellular bronchi and the first left intrapulmonary bronchus were dissected under binocular control. For isometric contraction experiments, rings about 3 mm in length were obtained from 1^st^, 2^nd ^and 3^rd ^airway generations, i.e., trachea, left and right extrapulmonary and left IPB. In order to avoid possible biases due to variation in ring size, contraction was normalised to a reference functional response (see below). When needed, the epithelium was mechanically removed.

### Preparation of human bronchial rings

Human bronchial rings were obtained from lung pieces collected for histological examination following resection for carcinoma. As in previous studies [17] specimens were selected from 15 patients whose lung function was within a normal range, i.e., whose forced expiratory volume in 1 second (FEV_1_) was above 80% of predicted. Quickly after resection, segments of human bronchi (3^rd ^to 5^th ^generation; 3–5 mm in internal diameter) were carefully dissected from a macroscopically tumour-free part of each of the histological pieces and transferred to the laboratory in an ice-cold PSS solution. Segments were then cut into rings measuring about 4–5 mm in length for isometric contraction measurements. Use of human tissues was performed according to national guidelines, in compliance with the Helsinki Declaration.

### Obtention of freshly isolated and cultured cells

For isolated cell-experiments, the muscular strip located on the dorsal face of the rat trachea was further dissected under binocular control. The epithelium-free muscular strip was cut into several pieces and the tissue was then incubated overnight (14 h) in low-Ca^2+ ^(200 μM) physiological saline solution (PSS; composition given below) containing 0.5 mg·ml^-1 ^collagenase, 0.35 mg·ml^-1 ^pronase, 0.03 mg·ml^-1 ^elastase and 3 mg·ml^-1 ^bovine serum albumin at 4°C. After this time, the muscle pieces were triturated in a fresh enzyme-free solution with a fire polished Pasteur pipette to release cells, which were collected by centrifugation. In control experiments, immunocytochemistry was performed using monoclonal mouse anti-smooth muscle α-actin antibodies and FITC-conjugated anti-mouse IgG antibodies to verify that the isolated cells obtained by dissociation were smooth muscle cells (data not shown).

For experiments on freshly isolated cells, cells were stored for 1 to 3 h to attach on glass coverslips at 4°C in PSS containing 0.8 mM Ca^2+ ^and used on the same day. For cell culture, coverslips with attached cells were placed in multiwell plates at 37°C in humidified air containing 5% CO_2 _in DMEM containing 0.5 U·mL^-1 ^penicillin, 0.5 mg·mL^-1 ^streptomycin and 0.25 μg·mL^-1 ^amphotericin B, and cultured in non-proliferating and proliferating conditions. For experiments in non-proliferating conditions, cells (15000 cells·mL^-1^) were cultured in the above-described DMEM supplemented with insulin, and ITS medium, which maintains the cells in quiescent state. For experiments in proliferating conditions, cells (7500 cells/mL) were cultured in the above-described DMEM supplemented with 10% foetal bovine serum. After 10 days, confluent cells were detached with a 0.5% trypsin-0.02% EDTA, resuspended and stored for 1 h to attach on coverslips at 4°C before use.

### Isometric contraction measurement

Isometric contraction was measured in isolate rings that were mounted between two stainless steel clips in vertical 5 ml organ baths of a computerized isolated organ bath system (IOX, EMKA Technologies, Paris, France) previously described [17]. Baths were filled with Krebs-Henseleit (KH) solution (composition given below) maintained at 37°C and bubbled with a 95% O_2_-5% CO_2 _gas mixture. The upper stainless clip was connected to an isometric force transducer (EMKA Technologies). Tissues were set at optimal length (Lo) by equilibration against a passive load of 1.5 g for extrapulmonary airways and 1 g for IPB. At the beginning of each experiment, supramaximal stimulation with acetylcholine (ACh, 10^-3 ^M final concentration in the bath) was administered to each of the rings to elicit a reference response. Rings were then washed with fresh KH solution to eliminate the ACh response. After the tension returned to baseline, the organ bath was filled with the appropriate solution, and unique or non-cumulative concentrations of agonists were added to the bath and the subsequent variation in tension recorded, and expressed as a percentage of the reference response to ACh in that ring. Each type of experiment was repeated for the number of rings from different specimens indicated in the text.

In epithelium-free experiments, the epithelium of isolated rings was rubbed using a plastic cylinder introduced in the lumen of the ring. Rings were frozen at the end of the experiment for histological examination of actual removal of the epithelium (data not shown).

### Fluorescence measurement and estimation of [Ca^2+^]_i_

[Ca^2+^]_i _responses of isolated tracheal myocytes were monitored fluorimetrically using the Ca^2+^-sensitive probe indo-1 as previously described [18]. Briefly, freshly isolated cells were loaded with indo-1 by incubation in PSS containing 1 μM indo-1 AM for 25 min at room temperature and then washed in PSS for 25 min. Coverslips were then mounted in a perfusion chamber and continuously superfused at room temperature. A single cell was illuminated at 360 ± 10 nm. Emitted light from that cell was counted simultaneously at 405 nm and 480 nm by two photomultipliers (P100, Nikon). [Ca^2+^]_i _was estimated from the 405/480 ratio using a calibration for indo-1 determined within cells.

ATP or ACh was applied to the tested cell by a pressure ejection from a glass pipette located close to the cell. No change in [Ca^2+^]_i _was observed during test ejections of PSS (data not shown). Generally, each record of [Ca^2+^]_i _response was obtained from a different cell. Each type of experiment was repeated for the number of cells indicated in the text.

### Solution, chemicals and drugs

Normal PSS contained (in mM): 130 NaCl, 5.6 KCl, 1 MgCl_2_, 2 CaCl_2_, 11 glucose, 10 Hepes, pH 7.4. Normal KH solution contained (in mM): 118.4 NaCl, 4.7 KCl, 2.5 CaCl_2_·2H_2_O, 1.2 MgSO_4_·7H_2_O, 1.2 KH_2_PO_4_, 25.0 NaHCO_3_, 11.1 D-glucose, (pH 7.4). In Ca^2+^-free solution, Ca^2+ ^was removed and 0.4 mM EGTA was added. In order to keep the osmotic pressure constant, in Na^+^-free solution, Na^+ ^was omitted and replaced by N-methyl-D-glucamine, and, for KCl-induced contraction, KCl was substituted to NaCl for the desired concentrations.

Collagenase (type CLS1) was from Worthington Biochemical Corp. (Freehold, NJ, USA). Bovine serum albumin, acetylcholine, carbachol, ATP, ATP-γ-S, α-β-methylene ATP, D600, RB2, H-89, caffeine and thapsigargin were purchased from Sigma (Saint Quentin Fallavier, France). Indo-1 AM was from Calbiochem (France Biochem, Meudon, France). Indo-1 AM and thapsigargin were dissolved in dimethyl sulphoxide which maximal concentration used in our experiments was < 0.1% and had no effect on the resting value of the [Ca^2+^]_i _(data not shown). DMEM, ITS, penicillin, streptomycin, amphotericin B and foetal bovine serum were from GIBCO-BRL (Invitrogen, Eragny-sur-Oise, France).

### Data analysis and statistics

Data are given as mean ± SEM. The maximal contraction *F*_*max *_was taken as the apparent maximal response, i.e., the response obtained with the maximal concentration used, even though the CRC had not reached a plateau. Overall differences in CRC were performed by ANOVA test. The transient effect of ATP was estimated by T_R10_, the time needed for the tension value to decrease to 10% *F*_*max*_, calculated from the maximal contraction. *F*_*max *_and T_R10 _were compared using Student's *t *tests. Statistical comparisons of [Ca^2+^]_i _response of isolated cells were carried out with Student's *t *tests for quantitative variables and χ^2 ^tests for qualitative variables. Results were considered significant at P < 0.05

## Results

### Effect of ATP on rat and human isolated airways

**Figure 1:**
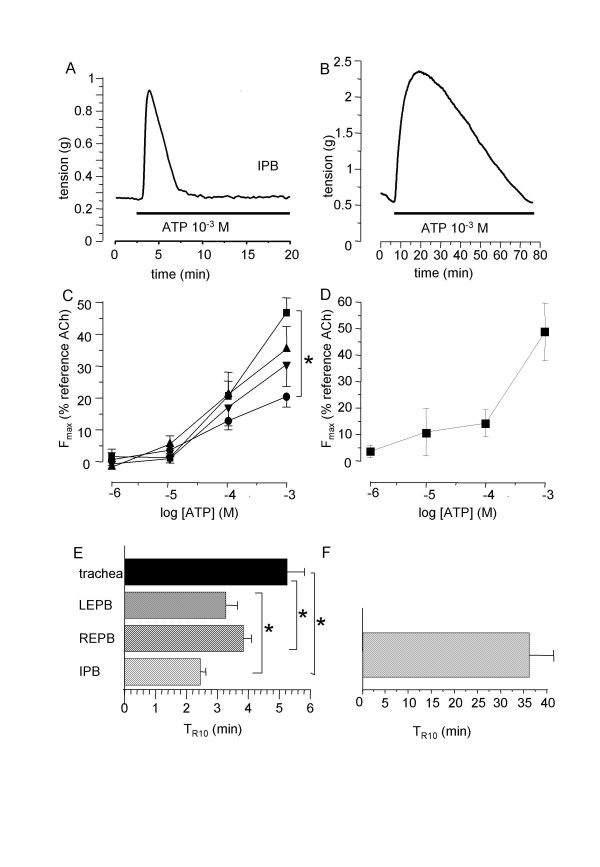
**Effect on ATP isolated airway rings**. A: typical trace of the effect of 10^-3 ^M ATP on rat IPB. B: typical trace of the effect of 10^-3 ^M ATP on human IPB. C: mean ATP-induced non-cumulative response curves in trachea (black circles) right EPB (down triangles), left EPB (up triangles) and left IPB (squares) from rat airways (n = 10). D: mean ATP-induced non-cumulative response curves in human IPB (n = 7). E: T_R10 _in rat trachea (black column) right (REPB) and left EPB (LEPB) (hatched columns), and left IPB (cross-hatched column). F: T_R10 _in human IPB (cross-hatched column) Error bars and SEM. *P < 0.05.

ATP induced a fast and transient contraction of rat isolated airway rings which amplitude depended on the concentration of agonist and the location along the airway tree. Original trace obtained in IPB is presented in figure 1A. Non-cumulative concentration-response curves, shown in figure 1C, indicated that the ATP-induced contraction was the greatest in IPB, and the lowest in trachea (n = 7 to 10). The time needed to return to baseline, expressed as T_R10_, is shown in figure 1E. As in rat airways, ATP induced a transient contractile response in human IPB, as illustrated by the original trace shown in figure 1B. The maximal response was in the same range as that observed in rat IPB (Figure 1D). However, the return to baseline was much slower in human bronchi (figure 1F) (n = 7).

### Effect of ATP on rat epithelium-free isolated airways

**Figure 2:**
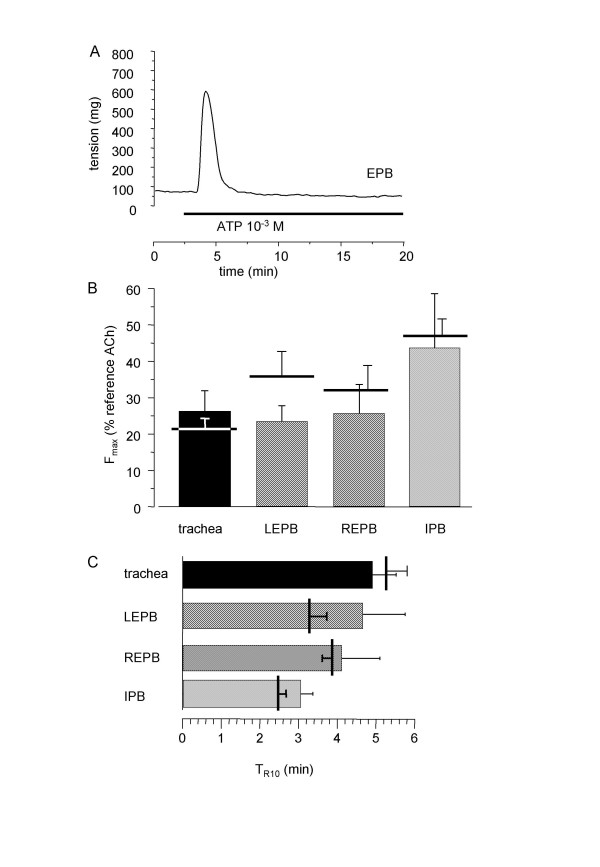
**Effect on ATP on rat epithelium-free isolated airway rings**. A: typical trace of the effect of 10^-3 ^M ATP on epithelium-free rat EPB. B: F_max _to 10^-3 ^M ATP in epithelium-free rings from trachea (n = 8), left and right EPB (n = 6), and left IPB (n = 7). Horizontal bars are F_max _in control rings. C: T_R10 _in rat trachea (black column) right and left EPB (hatched columns), and left IPB (cross-hatched column). Error bars are SEM. *P < 0.05.

In this set of experiments, for each rat, ATP was applied at fixed concentration (10^-3 ^M) on epithelium-denuded rings. Measurements were repeated on 6 to 8 specimens. The response pattern was similar to that obtained in intact rings (Figure 2A). Statistical comparison showed no difference between intact and epithelium-free rings, either on the maximal contractile response or on the return to baseline (figure 2B and 2C).

### Effect of ATP on freshly isolated and cultured tracheal myocytes

**Figure 3:**
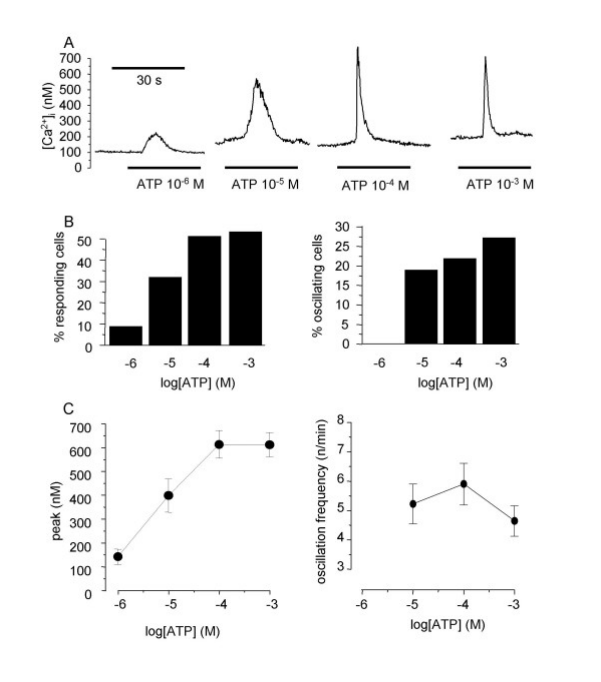
**Effect of ATP on freshly isolated rat tracheal myocytes**. A: original traces of the effect of several ATP concentrations (10^-6 ^to M 10^-3 ^M) on freshly isolated rat tracheal myocytes (n = 33 to 97 for each concentration). B: percentage of responding cells depending on ATP concentration (left panel) and percentage of oscillating responses in responding cells. C: abscissa: log concentration of ATP (M). Ordinates: amplitude of the Ca^2+ ^peak (left panel) in responding cells (left panel) and oscillation frequency in oscillating cells.

In a first set of experiments, ATP was applied at 10^-6 ^M (n = 33), 10^-5 ^M (n = 65), 10^-4 ^M (n = 97), and 10^-3 ^M (n = 82) on myocytes freshly isolated from rat trachea. Original representative [Ca^2+^]_i _responses are shown in figure 3A, and results are summarised in figure 3B, C. ATP stimulation resulted in a transient [Ca^2+^]_i _rise followed, in some cases, by several subsequent [Ca^2+^]_i _oscillations. The percentage of responding cells, the amplitude of the [Ca^2+^]_i _peak, and the percentage of oscillating responses were concentration-dependent. Similar experiments were performed with 10^-5^ACh (n = 61), a concentration that induces the maximal [Ca^2+^]_i _response [18]. The percentage of responding cells was 100%, the amplitude of the [Ca^2+^]_i _peak was 627 ± 30.2 nM, the percentage of oscillating response was 39.3%, and the frequency of oscillations was 7.83 ± 0.69 oscillations/min. Compared to the cholinergic response, the percentage of responding cells to 10^-3 ^M ATP and the frequency of oscillations were significantly lower, but not the amplitude of the peak nor the percentage of oscillating responses.

**Figure 4:**
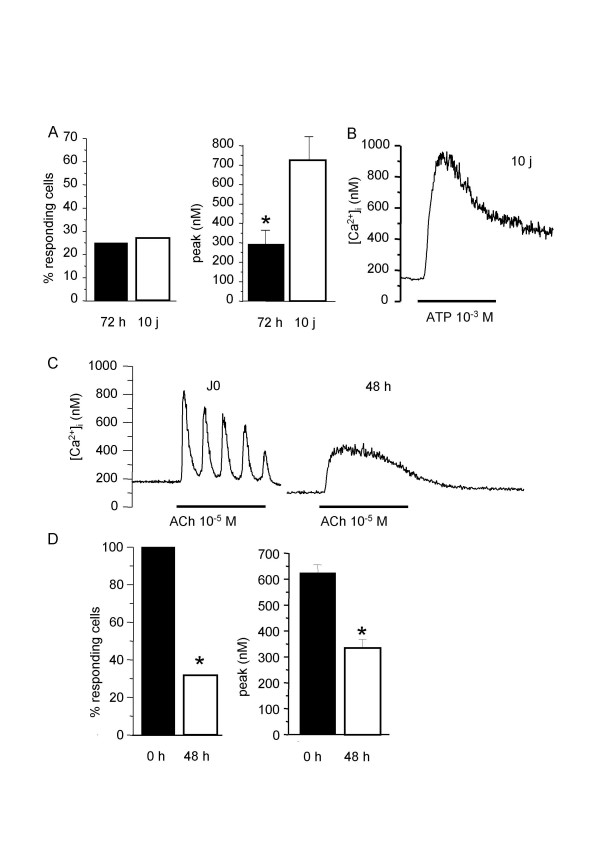
**Effect of ATP and ACh on cultured rat tracheal myocytes**. A: percentage of cells responding to 10^-3 ^M ATP, and amplitude of the [Ca^2+^]_i _peak, in cells cultured for 72 h in non-proliferating medium (black columns, n = 27) and in cells cultured for 10 days in proliferating medium (open columns, n = 35). B: typical single [Ca^2+^]_i _recording of a cell cultured for 10 days in proliferating medium stimulated with 10^-3 ^M ATP. C: typical single [Ca^2+^]_i _response to 10^-5 ^M ACh in tracheal myocytes freshly isolated (J0) (n = 61) and cultured for 48 h in non-proliferating medium (n = 26). D: percentage of cells responding to 10^-5 ^M ACh, and amplitude of the [Ca^2+^]_i _peak, in freshly isolated myocytes (black columns, n = 61) and in cells cultured for 48 h in non-proliferating medium (open columns, n = 26). *P < 0.05 *versus *responses in freshly isolated cells.

Since some authors have observed a [Ca^2+^]_i _response to ATP only in cultured cells [15], we investigated the [Ca^2+^]_i _response to 10^-3 ^M ATP in cells cultured for 3 days (n = 27) in non-proliferating medium and 10 days in proliferating medium(n = 35) (figure 4). Culture did not significantly alter the number of responding cells. 72 h-culture decreased the amplitude of the [Ca^2+^]_i _peak to ATP. In 10 day-cultured cells, the amplitude of the [Ca^2+^]_i _peak re-increased up to the values observed in non-cultured myocytes, and the general profile of the response dramatically altered, as shown in the original trace (figure 4B). To see whether the effect of cell culture on the [Ca^2+^]_i _response was specific to ATP, we compared the Ca^2+ ^response to ACh in cultured cells (n = 26) with that obtained in freshly isolated cells. After 2 days of culture in non-proliferating medium, the percentage of responding cells as well as the amplitude of the [Ca^2+^]_i _peak in responding cells were significantly reduced (figure 4C and 4D), and oscillating responses were only 12.5%.

### Role of intracellular Ca^2+ ^stores and extracellular Ca^2+ ^in ATP-induced response

**Figure 5:**
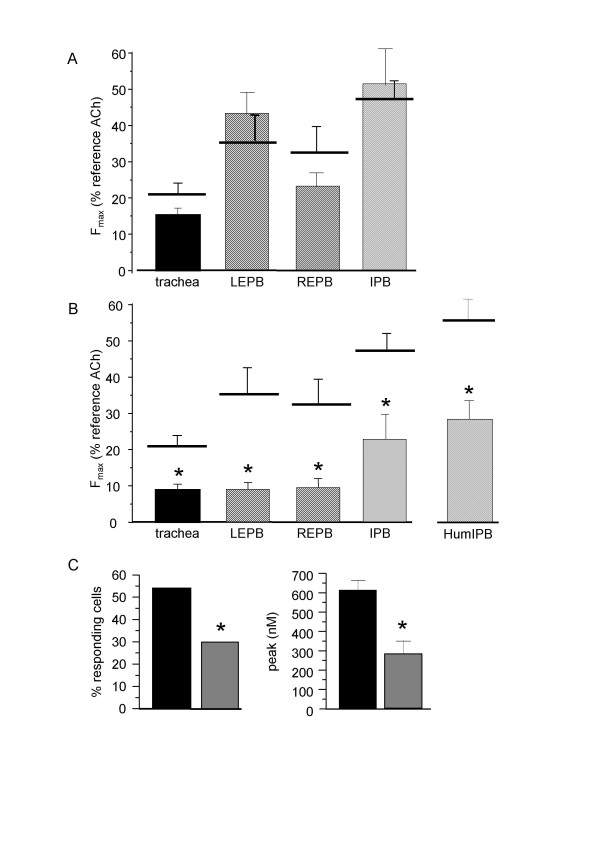
**Role of intracellular Ca^2+ ^stores and extracellular Ca^2+ ^in ATP-induced response**. A: F_max _to 10^-3 ^M ATP in rings from rat trachea (black column, n = 8) left (LEPB) and right (REPB) EPB (hatched columns, n = 8), and left IPB (cross-hatched column, n = 6) after depletion of intracellular Ca^2+ ^stores by application of thapsigargin and caffeine. Horizontal bars are F_max _in control conditions B: F_max _to 10^-3 ^M ATP rings from rat trachea (black column, n = 8) left (LEPB, n = 8) and right (REPB, n = 7) EPB (hatched columns), and left IPB (cross-hatched column, n = 8), and in human IPB (HumIPB, cross-hatched column, n = 5) in the absence of external Ca^2+^. Horizontal bars are F_max _in control conditions. C: percentage of rat freshly isolated tracheal myocytes responding to 10^-3 ^M ATP, and amplitude of the [Ca^2+^]_i _peak, in the presence (black columns, n = 61) and in the absence (grey columns, n = 30) of external Ca^2+^. Error bars are SEM. *P < 0.05.

In order to determine the implication of intracellular Ca^2+ ^stores in the response to ATP, we performed the following experiments: in the absence of extracellular Ca^2+^, rings from rats airways (n = 6 to 8) were exposed to 10^-6 ^M thapsigargin, an irreversible SERCA blocker. Ca^2+ ^release from the SR was triggered by 5 mM caffeine application for 30 min, followed by wash up. Such a protocol ensures the emptiness of the SR, which was verified by the fact that in these conditions, the contractile response to ACh, which has been shown to act via intracellular Ca^2+ ^release from the SR [18], is abolished (data not shown). After caffeine washout, Ca^2+ ^(2 mM) was reintroduced in the extracellular medium. Such a re-introduction did not change the basal tension (data not shown). 10^-3 ^M ATP was then applied to the tissues. As shown in figure 5A, the absence of intracellular Ca^2+ ^did not modify the ATP-induced contraction.

To assess the implication of external Ca^2+ ^influx in the response to ATP, we performed experiments on rat airways (n = 7 to 8) in the absence of extracellular Ca^2+^. In Ca^2+^-free KH solution, F_max _was significantly lower than in control conditions, and was below 10% of the ACh reference response, except in IPB where the remaining response, though significantly reduced, was above 20%. Similar experiments were performed on human IPB (n = 5). As in rat, the contractile response was significantly lower, but remained above 25%. Results are summarized in figure 5B.

Experiments in the absence of external Ca^2+ ^were also performed on freshly isolated tracheal myocytes (n = 30). Removal of extracellular Ca^2+ ^reduced both the percentage of responding cells to 10^-3 ^M ATP and the amplitude of the [Ca^2+^]_i _response in the responding cells, as shown in figure 5C, abolished [Ca^2+^]_i _oscillations.

### Role of L-type Ca^2+ ^channels and extracellular Na^+ ^in ATP-induced contraction

**Figure 6:**
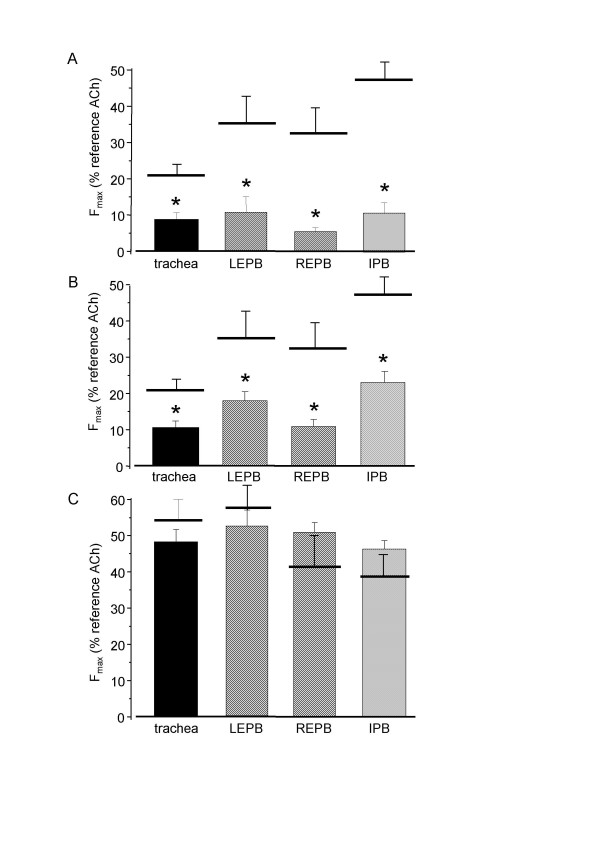
**Effect of D600 and extracellular Na^+ ^removal on ATP-induced response**. A: F_max _to 10^-3 ^M ATP in rat airway rings in the presence of 10 μM D600 (n = 7 to 10). B: F_max _to 10^-3 ^M ATP in rat airway rings in the absence of extracellular Na^+ ^(n = 7 to 8). C: F_max _to 30 mM KCl in rat airway rings in the absence of extracellular Na^+ ^(n = 5 to 7). Trachea: black column; left (LEPB) and right EPB (REPB): hatched columns; left IPB: cross-hatched column. Horizontal bars are F_max _in control conditions. Error bars are SEM. *P < 0.05.

Since ATP-induced response appeared to be dependent on extracellular Ca^2+^, we tested the effect of 10^-5 ^M D600, an inhibitor of the L-type voltage-dependent Ca^2+ ^channels on the contractile response to 10^-3 ^M ATP (n = 7 to 10). As shown in figure 6A, F_max _was significantly reduced in the presence of D600. In a following series of experiments, 10^-3 ^M ATP was applied to the rings in the absence of extracellular Na^+^. In these conditions, the ATP-induced response was significantly reduced in each type of rings, as shown in figure 6B (n = 7). By contrast, removal of extracellular Na^+ ^did not modify the contractile response to the depolarizing agent KCl (30 mM) (n = 5 to 7), as shown in figure 6C.

### Effect of α-β-methylene ATP and RB2 on ATP-induced contraction

**Figure 7:**
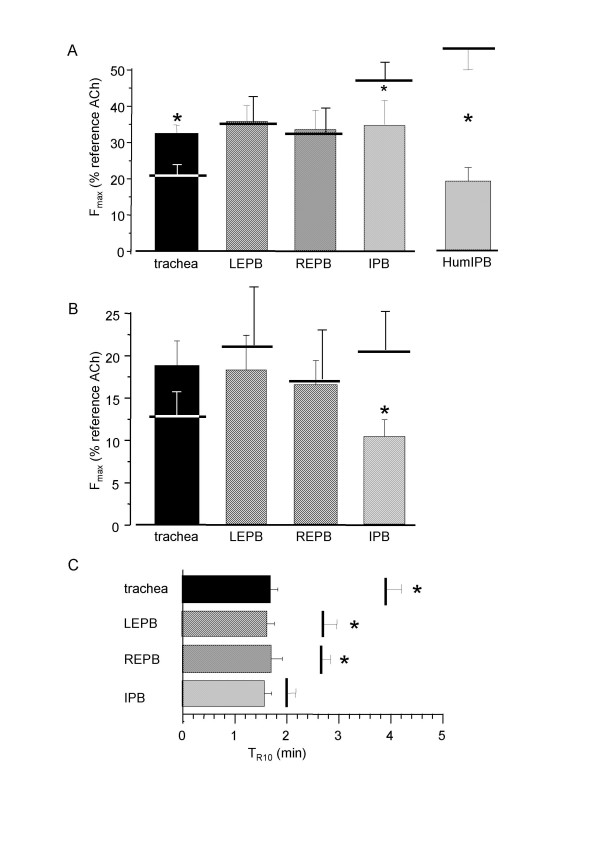
**Effect of RB2 and α-β-methylene ATP on rat airway rings**. A: F_max _to 10^-3 ^M ATP in rat airway rings (n = 8) and human IPB (HumIPB, n = 8) in the presence of 10 μM RB2. B: F_max _to 10^-4 ^M α-β-methylene ATP in rat airway rings (N = 7 to 8). Horizontal bars are F_max _in control conditions. C: T_R10 _in rat airway rings stimulated with 10^-4 ^M α-β-methylene ATP. Vertical bars are T_R10 _in control conditions, i.e., 10^-4 ^M ATP. Trachea: black column; left (LEPB) and right EPB (REPB): hatched columns; left IPB: cross-hatched column. Error bars are SEM. *P < 0.05.

In order to determine which type of P2 purinoreceptors was implicated in the contractile response to ATP, we tested the effect of RB2, a P2Y inhibitor, on the ATP-induced contraction and we measured the contractile response to α-β-methylene ATP, a specific agonist of P2X purinoreptors. Incubation with RB2 did not significantly modify the ATP-induced contractile response in extrapulmonary bronchi, but it significantly increased the response of trachea, and reduced that of IPB, (n = 10). RB2 also significantly reduced the contractile response of human IPB (n = 8). Results are shown in figure 7A. α-β-methylene ATP was used at 10^-4 ^M. As with ATP at the same concentration, the α-β-methylene ATP-induced contraction was transient. The amplitude of the contractile response was not different from experiments with ATP in similar conditions in extrapulmonary airways, but was significantly reduced in IPB (figure 7B). T_R10 _was significantly smaller in extrapulmonary airways, whereas it was not modified in IPB, as shown in figure 7C (n = 7 to 8).

### Effect of ATP-γ-S on rat isolated airways

**Figure 8:**
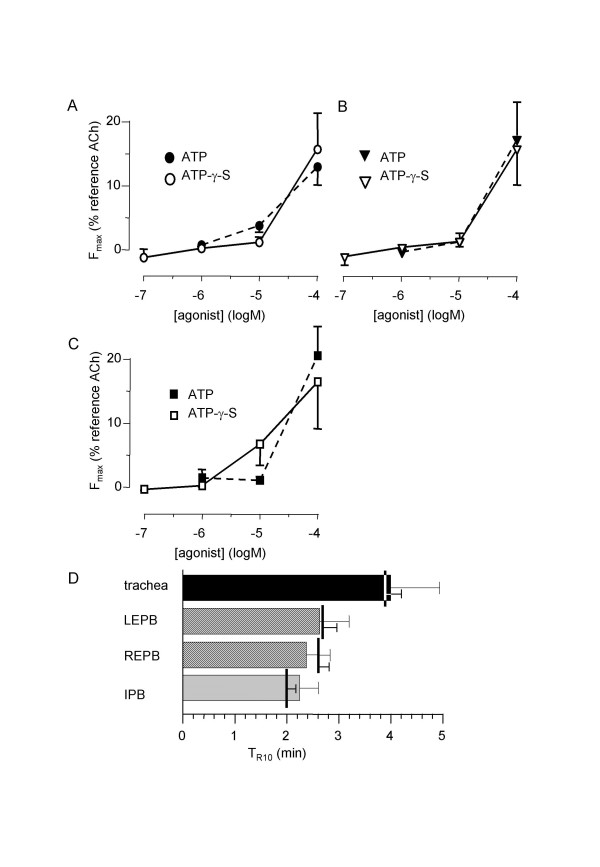
**Effect of ATP-γ-S on isolated airway rings**. A, B & C: mean ATP-induced (black symbols) and ATP-γ-S-induced (open symbols) non-cumulative response curves in trachea (A, n = 10) left EPB (B, n = 7), and left IPB (C, n = 10) from rat airways. D: T_R10 _in rat trachea (black column) right (REPB) and left EPB (LEPB) (hatched columns), and left IPB (cross-hatched column) stimulated by 10^-4 ^M ATP-γ-S. Vertical bars are T_R10 _in control conditions, i.e., 10^-4 ^M ATP. Error bars are SEM. *P < 0.05.

In order to evaluate a possible role of ATP degradation in the transience of the response, we assessed the effect of the non-hydrolysable ATP analogous, ATP-γ-S, from 10^-7 ^to 10^-4 ^M. Results are shown in figure 8. ATP-γ-S induced a fast and transient contraction which characteristics did not differ from that of ATP. The CRC were not significantly different from that obtained with ATP and neither was the T_R10 _(n = 5 to 10).

### Effect of indomethacin and H-89 on ATP-induced contraction in rat isolated airways

**Figure 9:**
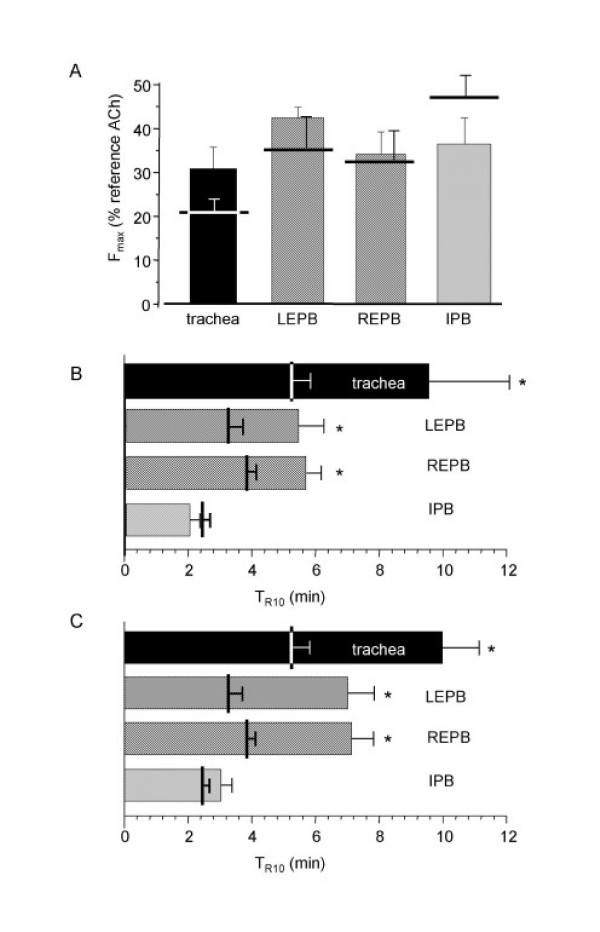
**Effect of indomethacin and H-89 on ATP-induced contraction in rat isolated airway rings**. A: F_max _to 10^-3 ^M ATP in rat airway rings in the presence of 10 μM indomethacin. Horizontal bars are F_max _in control conditions. B: T_R10 _in rat airway rings stimulated by 10^-3 ^M ATP in the presence of 10 μM indomethacin (n = 5 to 8) C: T_R10 _in rat airway rings stimulated by 10^-3 ^M ATP in the presence of H-89 (n = 8). Trachea: black column; left (LEPB) and right EPB (REPB): hatched columns; left IPB: cross-hatched column. Vertical bars are T_R10 _in control conditions. Error bars are SEM. *P < 0.05.

In order to identify a possible implication of arachidonic acid derivatives due to cyclooxygenase activity in the response to ATP stimulation, experiments were performed with 10^-5 ^M indomethacin. Rat tissues were incubated in the presence of indomethacin 30 min before ATP stimulation. The maximal contractile response was not significantly modified (figure 9A). By contrast, the return to baseline was significantly longer in the presence of indomethacin in extrapulmonary airways, but not in IPB (figure 9B). We tested the effect of H-89, an inhibitor of PKA, on the ATP-induced contraction. In the presence of H-89, T_R10 _was significantly increased in tracheal and extrapulmonary bronchial rings, but was not modified in IPB (figure 9C).

### Effect of successive ATP stimulations

**Figure 10:**
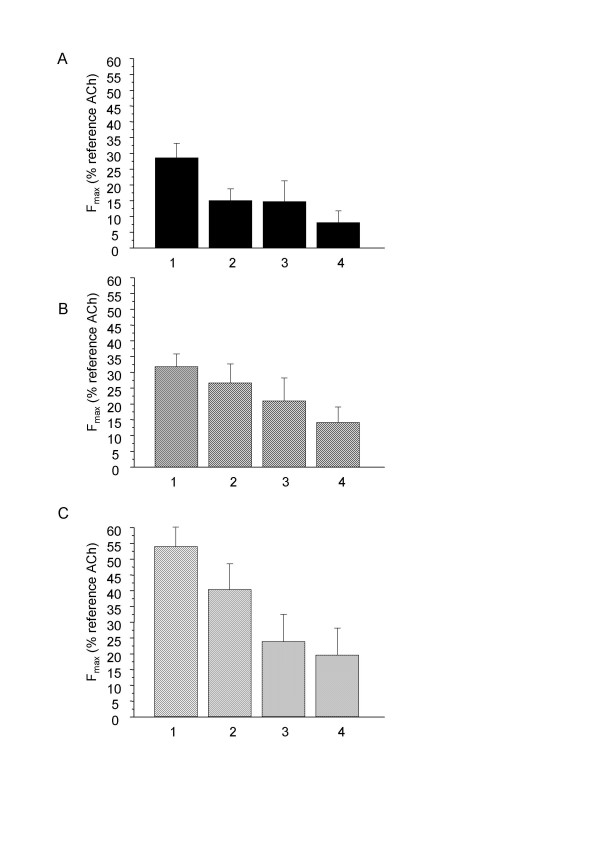
**Effect of successive ATP stimulation in rat isolated airway rings**. F_max _in response to 4 successive stimulations by 10^-3 ^M ATP at 15 min-intervals of rat trachea (A, n = 8) left EPB (B, n = 8), and left IPB (C, n = 8). Error bars are SEM.

In order to assess a possible desensitization of purinoreceptors that may explain the progressive return to baseline following the initial contraction, we performed 4 successive ATP stimulations. 10^-3 ^M ATP was applied for 5 minutes, then washed, and stimulations were performed at 15 minute-intervals. As shown in figure 10C, the maximal responses to successive stimulations were progressively decreased.

## Discussion

Our results showed that extracellular ATP induced a concentration-dependent transient contraction of rat and human airways, which both amplitude and mechanisms depend on the location along the airway tree. The ATP-induced response was not modified in the absence of epithelium, and mainly depended on the presence of external Ca^2+ ^and Na^+^. The response pattern was similar with the non-hydrolysable analogous ATP-γ-S.

The fact that extracellular ATP alone induced a transient contractile response in airways is in agreement with previous studies that have evidenced such a response profile in mouse IPB [8] and guinea-pig trachea [19,20], though due to different mechanisms. A biphasic contractile response has also been observed in other smooth muscles, such as vesical smooth muscle [21,22]. However, in rabbit trachea, Aksoy and co-workers failed to evidence any contractile effect of ATP alone in rabbit trachea, whereas, in human isolated bronchi, Finney and co-workers reported a small contractile effect of ATP on small airway preparation [23]. It appears then that the effect of extracellular ATP on airways depends both on the location along the airway tree and the species.

The contractile response observed in guinea-pig trachea has been reported, by some authors, to depend on the epithelium and/or related to arachidonic acid derivatives [19,20]. However, in rat airways including in trachea, we failed to evidence a significant involvement of the epithelium or the cyclooxygenase activity in the amplitude of the ATP-induced contractile response. Similarly, Bergner and co-workers concluded that in mouse IPB, ATP did not release sufficient quantities of prostaglandins to influence ATP-induced contraction [8]. The possible implication of epithelium-dependent prostanoid release in the ATP-induced response seems therefore to depend both on species and location alongside the airway tree.

Several studies performed on airway myocytes have shown that extracellular ATP induces [Ca^2+^]_i _increase [7,8,14-16]. We also found that direct exposure of isolated tracheal myocytes to ATP results in a concentration-dependent [Ca^2+^]_i _increase. Comparison of the response to ATP with that to cholinergic stimulation obtained in this study and in previous ones [18] indicates that the Ca^2+ ^response to ATP is smaller than that to ACh. Though the amplitude of the first peak is in the same range with the 2 agonists, the percentage of responding cells, as well as the percentage of oscillating responses and the frequency of oscillations was lower with ATP. This difference in the Ca^2+ ^response pattern explains why the contractile response to ATP is lower than that observed upon cholinergic stimulation.

We have demonstrated using both contraction measurements and [Ca^2+^]_i _recording in isolated cells that the major source of Ca^2+ ^was extracellular Ca^2+ ^influx, with an additional Ca^2+ ^release from internal stores, mainly in IPB, and, to a lesser degree, in extrapulmonary airways. These results are not in accordance with some previous studies that have shown that the ATP-induced response does not depend on extracellular Ca^2+ ^[8,14]. However, it should be noted that, in swine tracheal smooth muscle cells, the [Ca^2+^]_i _response to ATP stimulation appeared to depend on extracellular Ca^2+ ^[16]. These discrepancies may be due to different factors including species specificity. Also, the location along the airway tree may influence the relative participation of external versus internal Ca^2+^. Though removal of external Ca^2+ ^deeply reduced the contractile response to external airways, contraction of IPB remained significant even in the absence of extracellular Ca^2+^, a result in partial accordance with that of Bergner and co-workers [8]. Finally, results obtained on isolated cells may also differ between non cultured and cultured cells. Michoud and co-authors worked on cultured, not freshly isolated cells. Our experiments performed in both freshly isolated cells and cells cultured under several conditions indicated that cell culture, even primary culture, may alter not only the [Ca^2+^]_i _response to ATP but also to other agonists. This indicates that cell culture, even for short period, may critically modify the mechanisms responsible for Ca^2+ ^homeostasis in airway myocytes.

ATP-induced Ca^2+ ^influx is supposed to be due to Ca^2+ ^influx though P2X receptors. Surprisingly, in our study, the ATP-induced Ca^2+ ^response appeared to be dependent on L-type voltage-dependent Ca^2+ ^channels, indicating that [Ca^2+^]_i _increase was not due to a direct Ca^2+ ^influx through P2X receptors. However, P2X are not Ca^2+ ^specific and, hence, other cations may enter the cell through them. The fact that removal of extracellular Na^+ ^specifically inhibited the ATP-induced contraction, without altering the contraction elicited by direct depolarization by high extracellular K^+ ^concentration, indicates a functional coupling between ATP-activated channels and voltage-operated channels: Na^+ ^entry through ATP-activated channels may induce membrane depolarization and subsequent opening of voltage-operated channels and Ca^2+ ^influx. Such a coupling has been evidenced in PC-12 cells [24].

Taken together, our results about Ca^2+ ^sources are consistent with the activation of P2X receptors, associated, at least in IPB, with the activation of P2Y receptors. The specific P2X agonist α-β-methylene ATP induced a contractile response similar to that obtained with ATP. Moreover, the P2Y specific antagonist RB2 did not modify the response to ATP, except in IPB. Hence, the pharmacological characterization of the purinoceptors involved in the ATP-induced response seems in good accordance with the determination of the sources of [Ca^2+^]_i _implicated in the response.

The contraction induced by ATP is transient, with a return to baseline tension in several minutes. Previous studies have suggested that it can be ascribed to the degradation of ATP by ectonucleotidases [8]. Considering the CRC and T_R10_, return to baseline due to ATP degradation would require 99% ATP degradation in 3 to 6 minutes. Taking into account the size of a rat airway ring and the volume of the organ bath, such an explanation was highly improbable in our experimental conditions. This was confirmed by the fact that the contraction profile induced by ATP-γ-S, a non hydrolysable analogous of ATP, does not differ from ATP response. These results are in partial discordance with that obtained in mouse lung, where the response to ATP-γ-S was more prolonged than that to ATP [8]. However, according to the authors, although more prolonged than that obtained with ATP, the response to ATP-γ-S was transient.

Previous studies have shown an relaxant effect of ATP mediated by prostanoid release [25]. Such an effect does not seem to be involved in rat IPB, since the return to baseline was not modified by indomethacin. However, indomethacin did prolong the contractile effect of ATP in extrapulmonary airways, indicating that prostaglandin pathway is partially responsible for the transient contractile effect of ATP. Prostaglandin receptors EP_2 _have been identified in airway smooth muscle cells and their stimulation activates cAMP production and PKA activation [25,26]. Results obtained in the presence of the PKA inhibitor H-89, which, as indomethacin, significantly prolongs the contractile effect of ATP in trachea and EPB but not in IPB, show that in extrapulmonary airways, the transient contractile effect of ATP depends, at least in part, on PKA activation, probably due to prostaglandin receptor activation. An additional mechanism accounting for the transient contraction is the desensitization of the purinoceptors, since repeated stimulations resulted in a progressive decrease in the intensity of the response both in extra- and intrapulmonary airways. It is known that α-β-methylene ATP has a greater desentizating effect than ATP. The fact that, in trachea and EPB, α-β-methylene ATP-induced return to baseline was quicker than with ATP is in accordance with rapid P2X receptor desensitization in extrapulmonary airways. In IPB, where P2Y receptor activation is effective, the relaxant effect may be due to P2Y receptor desentization, a mechanism already evidenced in vesical smooth muscle [21]. However, in addition to PKA activation and/or receptor desensitization, other mechanisms may contribute to the transience of the ATP-induced contraction. Among them, opening of K^+ ^channels that have been identified as potential targets of purinoceptor activation may repolarize the plasma membrane and hence inhibit voltage-dependent Ca^2+ ^entry. In rat vascular smooth muscle, glibenclamide-sensitive K^+ ^channels have been shown to be implicated in the prolonged phase of ATP-induced vasorelaxation [27], whereas, in colonic smooth muscle cells, ATP appeared to activate Ca^2+^-dependent K^+ ^channels [28]. Very recently, a delayed ATP-elicited K^+ ^current, Ca^2+^- and glibenclamide-insensitive, has been identified in smooth muscle cells freshly isolated from rat aorta [29]. If present in ASM cells, these mechanisms may also contribute to the transience of the ATP-induced contraction.

Taken together, these results show regional variations in the effect of ATP along the airway tree, in terms of both amplitude of the response and underlying mechanisms. This suggests a segmental difference in the distribution of purinoceptor types and/or subtypes in the airways. On the basis of pharmacological studies, regional variation in P2 receptor expression has also been hypothesized in the pulmonary vasculature [30]. The expression of P2 purinoceptors has been investigated in several smooth muscle types, but few studies have been done in airway smooth muscle. Very recently, Govindaraju and co-workers, using RT-PCR and Western blotting, have identified in cultured human airway smooth muscle cell the expression of P2Y1, P2Y2, P2Y4 and P2Y6 receptor subtypes [31], but the authors did not investigate the possible expression of P2X receptors, whereas mRNA and protein expression of both P2X and P2Y have been evidenced in human vascular smooth muscle, P2X1, P2Y2 and P2Y6 being the predominant subtypes [32]. Data available in airway smooth muscle appear then to be fragmental, and systematic screening of P2 receptor expression along the airways requires further investigation.

## Conclusion

**Figure 11:**
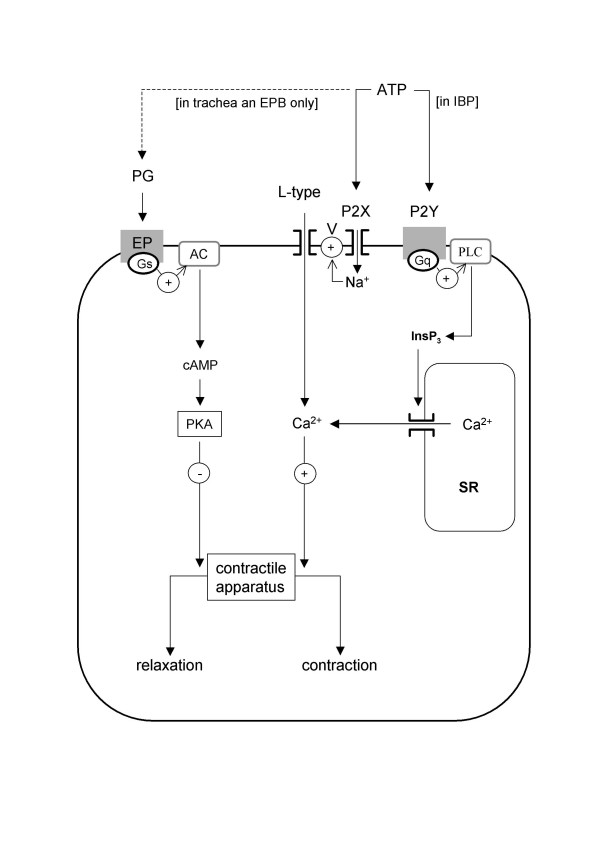
**Mechanisms of action of extracellular ATP on airway myocytes**. ATP opens P2X receptors, which triggers external Na^+ ^entry that depolarizes the plasma membrane and activates L-type voltage-operated Ca^2+ ^channels. The subsequent [Ca^2+^]_i _rises activates the contractile apparatus. In addition to these mechanisms, ATP acts on P2Y receptors and induces Ca^2+ ^release from SR via protein Gq and PLC activation, mainly in IPB. The progressive return to baseline following the initial contraction is due to desensitization of the purinergic receptors associated, in extrapulmonary airways, with epithelium-independent PG. PG binds to EP receptor coupled to protein Gs and AC and hence induces the production of cAMP, which inhibits the contractile apparatus via PKA activation.

In conclusion, we have shown that ATP has a transient contractile effect on human and rat airways, depending on the location along the airway tree. Based on our results in rat airways, we proposed the following mechanism for the effect of ATP on airways (figure 11): ATP acts directly on airway myocytes. Opening of P2X receptors triggers external Na^+ ^entry that depolarizes the plasma membrane and activates L-type voltage-operated Ca^2+ ^channels. The subsequent Ca^2+ ^influx is responsible for contraction. In IPB, in addition to these mechanisms, ATP acts on P2Y receptors and induces Ca^2+ ^release from intracellular Ca^2+ ^stores. The transient effect of ATP is not due to ATP degradation but can be attributed, as least partially, to purinoceptor desensitization and, in extrapulmonary airways, to PKA activation due to epithelium-independent prostaglandin release. Experiments in human IPB, though not as extensive as those performed in rat IPB, suggest that similar mechanisms are involved in human IPB.

## List of abbreviations

ACh: Acetylcholine

AC: Adenylcyclase

ASM: Airway Smooth Muscle

ATP: Adenosine triphosphate

[Ca^2+^]_i_: cytosolic Ca^2+ ^concentration

cAMP: Cyclic adenosine monophosphate

CRC: Concentration-Response Curve

CLS: Collagenase

DMEM: Dulbecco's modified Eagle's medium

D600: Methoxyverapamil

EDTA: Ethylene diamine tetra-acetic acid

EGTA: Ethylene glycol tetra-acetic acid

EPB: Extrapulmonary bronchi

*F*_*max*_: Maximal apparent contraction

IPB: Intrapulmonary bronchi

FEV: Forced expiratory volume

ITS medium: Insulin, transferrin and selenite medium

Indo-1 AM: Indo-1 acetoxymethylester

KH: Krebs-Henseleit

PLC: Phospholipase C

PKA: Protein kinase A

PSS: Physiological saline solution

PG: Prostaglandin

RB2: Reactive blue 2

SERCA: SarcoEndoplasmic Reticulum Ca^2+ ^ATPase

SR: Sarcoplasmic Reticulum

T_R10_: time needed for the tension value to decrease to 10% *F*_*max*_

## Competing interests

The author(s) declare that they have no competing interests.

## Authors' contributions

BM carried out the contractile experiments and [Ca^2+^]_i _recording on isolated cells, participated in the analysis of the data, and helped the draft of the manuscript. RM participated in the design of the study and helped the draft of the manuscript. ER conceived the study, participated in its design, helped in [Ca^2+^]_i _recordings, performed statistical analysis and drafted the manuscript.
